# Tibial Anterior Cruciate Ligament Avulsion Fractures in Pediatric and Adult Populations: A Systematic Literature Review

**DOI:** 10.3390/jcm14176316

**Published:** 2025-09-07

**Authors:** Vincent Landré, Michel Teuben, Felix Karl-Ludwig Klingebiel, Alba Shehu, Falko Ensle, Hans-Christoph Pape, Thomas Rauer

**Affiliations:** 1 Department of Traumatology, University Hospital Zurich, University Zurich, Raemistrasse 100, 8091 Zurich, Switzerland; 2 Institute for Diagnostic and Interventional Radiology, University Hospital Zurich, 8091 Zurich, Switzerland; 3 Sports Knee Institute, 5000 Aarau, Switzerland

**Keywords:** tibial eminence fracture, tibial spine avulsion, adult and pediatric population

## Abstract

**Objectives:** Tibial anterior cruciate ligament avulsion fractures (TAFs) are avulsions of the anterior cruciate ligament (ACL) from its insertion at the tibial intercondylar eminence that share the same trauma mechanism as ACL tears. TAFs were initially considered to be a pediatric equivalent to adult ACL ruptures due to the weaker insertion of the ACL on the immature tibial spine. Recent literature suggests that adult TAFs may be more common than previously thought. The incidence, possible concomitant injuries, and other differences between pediatric and adult TAFs remain a topic of ongoing debate in the literature. This systematic review provides a descriptive synthesis of the symptoms, biomechanics, and treatment outcomes of TAFs in pediatric and adult populations. This study highlights notable trends but avoids formal comparisons or meta-analysis due to heterogeneity in the literature. **Methods:** A systematic review was conducted on human-related studies involving tibial anterior cruciate ligament avulsion fractures, identified in PubMed^®®^ and EMBASE^®®^ databases between 2000 and 2024. Studies in English or German were included, while editorials, reviews, experimental studies, and papers with insufficient data were excluded. Data were extracted on patient demographics, trauma mechanisms, fracture classification, diagnostic modalities, treatment approaches, and clinical outcomes. Specific outcome parameters included: incidence and type of postoperative complications, return to sport rate, revision surgeries, hardware removal rates, and duration of follow-up. Due to heterogeneity in reporting, a descriptive synthesis approach was used rather than a meta-analysis. **Results:** The systematic search identified 3938 publications, with 2707 articles screened after duplicate removal. A total of 56 studies met the inclusion criteria. A total of 677 tibial avulsion fractures (TAF) were analyzed, with 208 (30.4%) pediatric and 469 (69.6%) adult patients. Type III fractures were most common in both groups (pediatric: 63.9%, adult: 63.4%). Concomitant injuries were more frequent in adults (35.6%) than children (8.2%). Arthroscopic surgery was the predominant technique (pediatric: 79.1%, adult: 87.8%). Fixation methods differed: pediatric cases more often used screws (40.5%) and sutures (38.2%), while adults favored sutures (49.7%) and suture anchors (23.1%). Complications were more frequent in pediatric patients (35.1% vs. 17.1%). **Conclusions:** TAFs show age-related differences in injury patterns and outcomes. Pediatric cases are mostly sports-related, while adult cases are commonly due to road traffic accidents. Concomitant injuries are more frequent in adults, whereas pediatric patients experience higher rates of arthrofibrosis and instability. Adults are more prone to malunion and non-union. These findings support the need for age-specific diagnostic and treatment strategies.

## 1. Introduction

Tibial anterior cruciate ligament avulsion fractures (TAF) are osseous avulsions of the anterior cruciate ligament from its anatomical insertion at the intercondylar eminence of the tibia [1]. Unlike ACL tears, which involve disruption of the ligament fibers themselves, TAFs occur when tensile forces on the ACL result in a fracture of the tibial eminence, often leaving the ligament intact but attached to an avulsed bony fragment [2]. Although more prevalent in children, TAFs remain rare, with an incidence of about 3 per 100,000 annually [3]. They share a similar mechanism of injury with anterior cruciate ligament (ACL) tears [4]. In pediatric patients, the incomplete ossification of the tibial eminence and relatively stronger ligaments make bony avulsions more likely than ACL tears [5]. In contrast, adults tend to experience ligamentous injuries due to changes in bone density and ligament elasticity with age [6]. TAFs frequently occur during sports activities involving sudden deceleration, pivoting, or landing from a jump, such as soccer, basketball, and skiing. Associated injuries like meniscal tears, collateral ligament sprains, or chondral damage can complicate the clinical picture and influence treatment decisions. Prompt and accurate diagnosis is essential to avoid long-term complications including knee instability, stiffness, or growth disturbances in skeletally immature patients. In 1959, Meyers and McKeever described three types of TAFs. Type I fractures are nondisplaced. Type II fractures involve displacement of the anterior margin, while type III fractures are completely displaced. Zaricznyj introduced a type IV fracture, characterized by a comminuted fragment [7]. Recent adaptations incorporate advanced imaging, especially MRI, to improve diagnostic precision [8]. When diagnosing TAFs a magnetic resonance imaging-based system reduces grading ambiguity and can clarify the appropriate treatment [9]. Moreover, the integration of imaging data with arthroscopic findings allows clinicians to more accurately assess ligament tension, fragment reducibility, and joint integrity, which in turn influences surgical planning. The role of MRI is especially critical in pediatric patients, where associated cartilaginous and soft tissue injuries may not be visible on plain radiographs. This highlights the need for a standardized diagnostic algorithm that incorporates MRI as a primary tool, particularly in cases of suspected concomitant pathology. Additionally, recent interest in 3D imaging and CT-based modeling has improved preoperative planning in complex or comminuted fractures, particularly in adult populations. These technologies not only enhance visualization but may also support the development of patient-specific surgical guides and implant strategies in the future. Nondisplaced type I fractures are usually treated with immobilization, pain management and physical therapy until radiographic evidence of bone union is established [10]. The optimal treatment for type II TAFs is still a subject of debate. Initial attempts at closed reduction can be made; however, if this proves insufficient, arthroscopic or open reduction and fixation are advised [9]. Type III and IV TAFs, characterized by complete displacement or comminution, necessitate surgical intervention [11]. Emerging surgical techniques, such as arthroscopic suture fixation or bioabsorbable screw placement, aim to optimize outcomes while minimizing surgical morbidity. Postoperative rehabilitation protocols also play a crucial role in functional recovery. Previous studies have examined TAFs collectively, but age-specific differences in presentation, biomechanics, treatment, and outcome remain underexplored. This systematic literature review aims to describe the characteristics and management strategies of TAFs in pediatric and adult populations, identifying trends and gaps in the current literature rather than conducting direct statistical comparisons.

## 2. Material and Methods

According to the PRISMA guidelines [12], a comprehensive systematic search was conducted between 1 October and 22 December 2024, in PubMed^®^ and EMBASE^®^ databases for studies published between 1 January 2000, and 22 December 2024. The search targeted human studies focused on fractures of the eminentia intercondylaris (tibial eminence) associated with the anterior cruciate ligament (ACL). The search terms and MeSH headings used can be found in the Supplementary Materials. Only peer-reviewed human studies published in English or German were eligible. Systematic reviews, meta-analyses, case reports, in vitro/in vivo experimental studies, editorials, conference abstracts, and studies with insufficient data reporting were excluded. All study designs with a minimum of clinical detail were included, regardless of level of evidence, as long as they provided relevant outcome data on treatment strategies for tibial eminence fractures. The included articles were then screened in full text for relevant information by two independent reviewers. Discrepancies in inclusion were resolved through discussion or consultation with a third reviewer to minimize selection bias. The following outcome parameters were extracted from the included studies: incidence and type of postoperative complications (including arthrofibrosis, instability or laxity, pain, malunion, and non-union), rate of revision surgeries, rate of hardware removal, long-term return to sport, and duration of follow-up. Complications were defined as any adverse clinical findings reported during follow-up that required intervention or impacted function. Revision surgery was defined as any secondary procedure related to the initial TAF treatment. Hardware removal included planned or unplanned extraction of implants. Return to sport was assessed as the proportion of patients who resumed physical activity post-treatment. Follow-up duration was recorded in months to assess the length of clinical monitoring. Outcomes were reported separately for pediatric and adult populations where available. In cases where age-based differentiation was not provided in the original study, attempts were made to contact authors or infer classification based on study context and participant demographics. Data were extracted using a pre-defined form to ensure consistency and reduce data entry errors. Statistical or meta-analytic techniques were not applied due to considerable heterogeneity in patient populations, outcome measures, surgical techniques, and reporting standards across studies. Therefore, a descriptive and narrative synthesis approach was adopted to summarize findings qualitatively. A structured data extraction process was employed, and study quality was evaluated based on reporting completeness and outcome clarity. A formal risk of bias assessment was not performed due to the heterogeneity of study designs, which is acknowledged as a limitation. However, variations in follow-up duration and inconsistencies in complication definitions were considered when interpreting findings. Due to the significant heterogeneity in study designs, outcome definitions, and reporting standards across the included studies, we did not perform a meta-analysis or apply statistical models to formally combine results. Instead, findings are presented as descriptive summaries and crude proportions to provide an overview of trends across pediatric and adult populations.

## 3. Results

**Figure 1:**
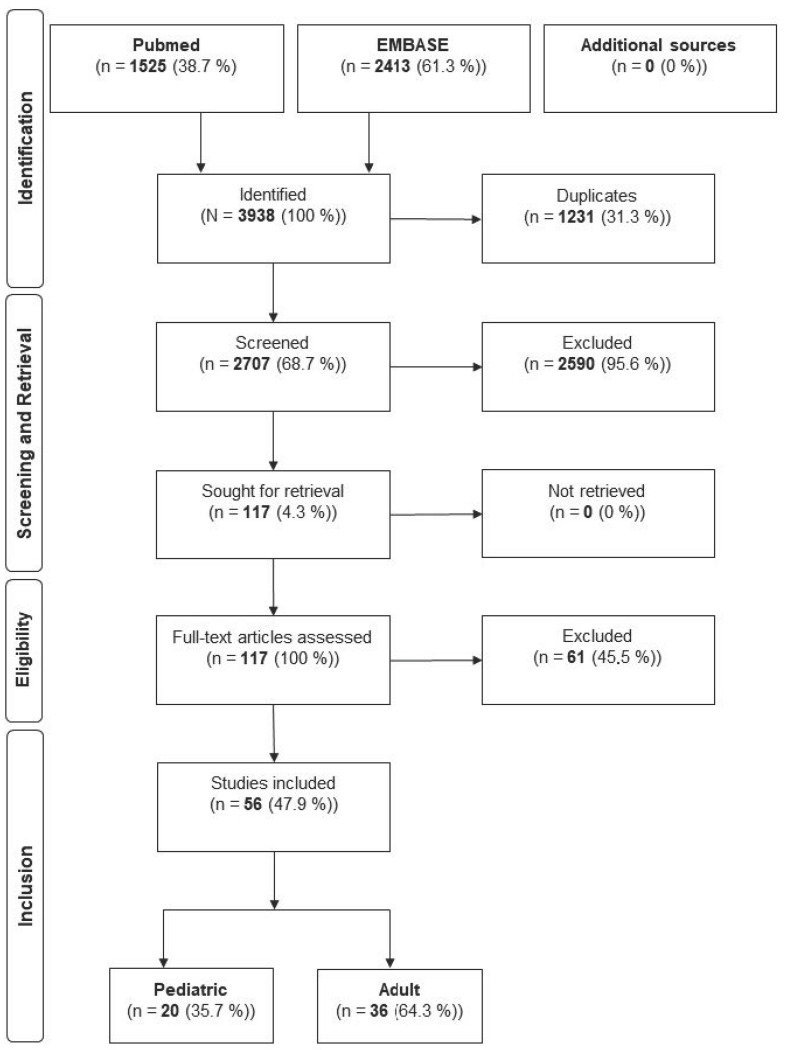
Flowchart of the literature Analysis.

The initial systematic search using MeSH criteria yielded 3938 publications. After removal of duplicates, 2707 articles were screened, and 56 articles met the inclusion criteria and have therefore been included (Figure 1). The reported numbers represent unadjusted pooled counts and proportions from the included studies. These values should be interpreted as descriptive indicators of observed patterns, rather than statistically validated comparative estimates (Table 1).

Table 2 contains a summary of the concomitant skeletal and soft tissue injuries in association with TAF.

Table 3 presents a comparison of the mechanism of injury of TAF between the pediatric and the adult population.

Table 4 highlights the diagnostics and treatment in adult and pediatric patients who suffered a TAF.

Table 5 reports the outcome and complications of the treatment of adult and pediatric patients who suffered a TAF.

## 4. Discussion

The current systematic literature review describes differences in the occurrence of TAF between pediatric and adult populations in terms of age, injury mechanisms, and possible skeletal maturity, without establishing causality. Given the heterogeneity of the included studies, our results should be interpreted as descriptive trends rather than formal statistical comparisons. Although this limits precision, the structured synthesis highlights clinically relevant age-specific patterns without introducing potentially misleading pooled effect estimates. In this respect, our study has yielded the following main results:

1. While TAFs are more common in males overall, the underlying mechanism in the pediatric population is usually sports trauma, whereas the underlying mechanism in the adult population is usually a road traffic accident.
2. Across the reviewed studies, concomitant injuries were more often reported in adult patients than in pediatric patients, although differences may also reflect study design and reporting variability.
3. Complications in general and arthrofibrosis, pain and revision surgery in particular are more common in the pediatric population, while malunion and non-union are more common in adults.

TAF are rare injuries. They can appear both in the pediatric and adult population [13]. The higher incidence in children is explained by the stronger ligament compared to the weaker bone and growth plate, where ACL fibers are firmly attached to the epiphysis [14,15]. Usually, male patients are more likely to be affected by a TAF [16]. This is mainly influenced by three factors. Previous biomechanical studies have suggested that increased quadriceps force may contribute to tibial spine avulsion risk, particularly in males [17]. Secondly, male individuals are more likely to engage in sports during adolescence which also increases the risk of suffering a TAFs [18]. Lastly, physiodesis of the proximal tibial tubercle occurs at later ages in male individuals, which increases the likelihood of a TAF in the pediatric population [5]. This gender-based discrepancy in injury risk may be multifactorial and highlights the importance of understanding behavioral and physiological differences between sexes. As males often participate in more high-intensity or contact sports during adolescence, they are exposed to repetitive stress and acute trauma to the knee joint [19]. In addition, differences in muscle mass distribution, quadriceps-to-hamstring strength ratio, and hormonal influences may also contribute to this increased susceptibility [20]. Biomechanical studies showed that TAFs are influenced by age and skeletal maturity [21,22]. Moreover, the diagnostic delay often seen in pediatric cases can be attributed to non-specific symptoms and lower clinical suspicion, especially when initial radiographs appear unremarkable. This underlines the importance of maintaining a high index of suspicion and incorporating advanced imaging techniques when clinical signs persist despite inconclusive X-rays. Timely recognition and intervention are crucial to avoid complications such as joint instability, chronic pain, and early onset osteoarthritis. If an TAF with possible concomitant injuries is not timely diagnosed, it can result in poor clinical outcomes and lead to early degenerative changes in the knee [23]. TAFs commonly occur in sports due to a sudden, forceful contraction of the quadriceps muscle, which exerts a pulling force on the tibial tuberosity via the patellar tendon [24]. This can happen indirectly through eccentric contractions, where the quadriceps work to resist knee flexion during landing, or directly through concentric contractions that generate strong knee extension [25]. In adults, TAFs are more commonly linked to high-energy trauma, including motor vehicle collisions, which result in more complex and comminuted fracture patterns. These mechanisms reflect the biomechanical differences between immature and mature skeletons, where the relatively brittle adult bone may fracture instead of avulsing. As a result, adults often present with more severe concomitant injuries including meniscal tears, ACL ruptures, and cartilage damage, further complicating diagnosis and treatment. To achieve effective management and determine the optimal treatment strategy appropriate, imaging, including X-Ray and computed tomography (CT), to assess the size of the avulsion fragment and the degree of comminution, and MR-Imaging, to identify concomitant injuries, should be performed [26]. Early and comprehensive imaging is crucial not only for diagnosis but also for surgical planning. CT offers the superior delineation of fracture fragment geometry, while MRI provides detailed information on soft tissue and ligamentous structures [27]. Unfortunately, only 35% of pediatric studies reported MRI use, suggesting a potential underutilization of advanced imaging in this cohort. This may result in missed diagnoses of associated injuries such as meniscal tears or cartilage lesions, which are clinically relevant for treatment planning and prognosis. However, our review identified a lack of standardization in the diagnostic approach across studies, with variability in imaging modalities used, potentially leading to the underreporting of associated injuries. Recommendations for treatment vary between countries and institutions. Treatment depends on the severity and extent of fracture displacement. It varies from immobilization, closed reduction followed by immobilization, open reduction with fixation, and arthroscopic reduction with fixation [28]. In general, Meyers and McKeever Type I injuries are treated with a conservative strategy. For more severe injuries, a surgical response is favored, but varies between institutions [10]. In general, arthroscopic transosseous suture fixation is the most common treatment, as it provides stable fixation at the base of the ACL and reduces the need for subsequent surgery, such as a second operation to remove implants after screw fixation [29]. This method also minimizes disruption to the growth plate, making it particularly suitable for pediatric patients. Transepiphyseal fixation, although technically effective, poses a significant risk of growth disturbance and is therefore discouraged in skeletally immature individuals. Fixation using non-absorbable sutures is favored for its lower complication rates, reduced risk of hardware irritation or failure, and elimination of secondary procedures. This is especially important in pediatric cases to avoid permanent intra-articular metallic hardware and mechanical disturbance of the fixation devices [30,31]. In pediatric cases, it can be advantageous, if necessary at all, to use a small paramedial arthrotomy to reduce and fix the fragments due to the adapted size of the instruments to limit the size of the knee joint [32]. Transepiphyseal fixation is not recommended in the pediatric population since it bears the risk of anterior growth arrest and hyperextension deformity of the knee [29]. In the literature, arthrofibrosis also remains the most common complication, with a high variation ranging from 10% to 29% [33]. Risk factors include delayed surgery, a longer operative time and delayed mobilization [33]. Pediatric patients are more prone to arthrofibrosis, likely due to longer periods of immobilization and heightened inflammatory responses. Additionally, surgical access in smaller joints can be more technically demanding, possibly resulting in greater tissue trauma. This suggests that early physical therapy and structured rehabilitation protocols should be universally implemented, particularly in pediatric patients, to mitigate risk. Yet, our review revealed inconsistency in postoperative management, further emphasizing the need for consensus-based rehabilitation guidelines. Emphasis should be placed on early motion protocols and regular physiotherapy to reduce the risk of postoperative stiffness. In cases of non-union or delayed union fractures, treatment using open reduction or arthroscopic techniques are considered as safe and effective [32]. Using nonabsorbable sutures for osteosynthesis is suggested to prevent the fragmentation of the intercondylar eminence, avoid hardware impingement, reduce the risk of neurovascular and intra-articular injuries, and eliminate the need for a second surgery to remove hardware [34]. In contrast to the pediatric group, adults demonstrate higher rates of non-union and malunion, which may be attributed to comorbidities like smoking, diabetes, or osteoporosis. These factors can impair bone healing and lead to prolonged disability. Close radiographic follow-up is essential in this group to detect healing complications early and consider intervention before chronic instability or arthritis develops. Some authors suggest that a better outcome correlates with the young age for patients after an arthroscopic fixation [35]. This review is limited by significant heterogeneity among included studies in terms of design, treatment strategies, outcome measures, and reporting quality, which prevented statistical comparison and required a descriptive approach. Inconsistent data reporting and varying follow-up durations may have introduced bias, particularly in complication rates. No formal risk of bias assessment was performed, and overlapping study populations cannot be entirely excluded. Another limitation is the absence of formal statistical synthesis or risk-of-bias assessment, which reduces the ability to directly compare outcomes across subgroups and may affect the generalizability of the reported frequencies. These limitations highlight the urgent need for prospective, multicenter trials utilizing standardized outcome reporting tools and follow-up intervals. Establishing uniform diagnostic and treatment protocols will enable more meaningful comparisons and help clarify the best management practices for different age groups. These factors limit the generalizability of the findings and highlight the need for standardized, prospective research in this area. In conclusion, distinct patterns were identified between pediatric and adult tibial anterior cruciate ligament avulsion fracture cases, particularly in relation to injury mechanisms, complications, and treatment approaches. However, these observations should be interpreted within the context of a descriptive synthesis. The absence of standardized outcome measures and the heterogeneity among the included studies limited the potential for rigorous statistical comparison. Effective management of TAF prompts appropriate imaging including CT and MR-Imaging, to identify associated injuries. Preferred treatment strategies vary between institution and depend mainly on fracture type and severity, concomitant injuries and patient age. Arthroscopic transosseous suture fixation was most frequently described in the included studies, particularly for pediatric patients. While some authors advocate it for its stability and reduced need for hardware removal, these findings should be interpreted as trends rather than definitive evidence of superiority. Pediatric patients face higher rates of complications like arthrofibrosis, while adults have more mal- and non-union issues, suggesting the need for age-specific treatment approaches. Ultimately, the management of TAF should be patient-centered, age-informed, and guided by fracture morphology and associated injuries. While advancements in arthroscopic techniques and fixation materials have improved outcomes, complications remain significant and vary by age group. Pediatric patients require careful surgical planning to avoid growth disturbances, while adults demand vigilant follow-up to detect healing issues. Future research must focus on high-quality evidence generation to define best practices, improve outcomes, and minimize complications in both age groups. A limitation of this review is that the search strategy was not registered in a public protocol repository. This omission may reduce methodological transparency and increase the risk of reporting bias. Future systematic reviews on this topic should consider prospective protocol registration to strengthen rigor and reproducibility.

## Figures and Tables

**Table 1:** Demographics and Meyers & McKeever classification of the TAF.

Characteristics	Pediatric n (%)	Adult n (%)
n	208 (30.4)	469 (69.6)
Gender		
Male	126 (60.6)	241 (62.9)
Female	69 (39.4)	142 (37.1)
Not reported	13	86
Meyers and McKeever		
Type I	4 (2.2)	3 (0.8)
Type II	60 (32.8)	87 (23.8)
Type III	117 (63.9)	232 (63.4)
Type IV	2 (1.1)	44 (12)
Not reported	25	103

**Table 2:** Concomitant skeletal and soft tissue injuries.

Type of Injury	Pediatric n (%)	Adult n (%)
n	8 (8.2)	100 (35.6)
Meniscus injuries	8 (100)	56 (19.9)
Other ligament injuries	0	24 (8.5)
Osteochondral injuries	0	32 (11.4)
Joint dislocations	0	1 (0.4)
Not reported	110	188

**Table 3:** Mechanism of injury.

	Pediatric n (%)	Adult n (%)
Sports	94 (65.7)	97 (27.6)
RTA with a car	19 (13.3)	103 (29.3)
RTA with a motor- or bicycle	16 (11.2)	106 (30.2)
Fall	14 (9.6)	45 (12.9)
Not reported	65	118

**Table 4:** Diagnostics and treatment.

	Pediatric n (%)	Adult n (%)
Diagnostics		
MRI performed	7 out of 20 Authors (35)	19 out of 36 Authors (52.8)
Surgical technique		
Conservative	10 (7.2)	5 (1.1)
Arthroscopic	110 (79.1)	411 (87.8)
Open	19 (13.7)	53 (11.1)
Not reported	69	1
Fixation method		
Screws	70 (40.5)	29 (6.6)
Suture	66 (38.2)	219 (49.7)
Suture anchors	22 (12.7)	102 (23.1)
Other	15 (8.7)	91 (20.6)
Not reported	35	28

**Table 5:** Outcome and complications.

	Pediatric n (%)	Adult n (%)
Period to last Follow-Up *	50.5 months (SD 90.6)	25 months (SD 39.1)
Revisions	55 (26.4)	74 (18.6)
Hardware removal	33 (15.9)	62 (15.6)
Long-term return to sport	96 out of 99 (97)	202 out of 233 (86.7)
Complications	73 (35.1)	80 (17.1)
Arthrofibrosis	42 (20.2)	31 (6.6)
Instability & Laxity	23 (11.1)	19 (4.1)
Malunion	4 (1.9)	11 (2.3)
Nonunion	1 (0.5)	15 (3.2)
Pain	3 (1.4)	4 (0.9)
Not reported	2 out of 20 Authors (10)	0 out of 36 Authors

* Standard deviation (SD).

## Supplementary Materials

The following supporting information can be downloaded at: https://www.mdpi.com/article/10.3390/jcm14176316/s1, Search terms and MeSH-headings.

## Abbreviations

ACL	Anterior Cruciate Ligament
CT	Computed Tomography
MeSH	Medical Subject Headings
MRI	Magnetic Resonance Imaging
ORCID	Open Researcher and Contributor ID
PRISMA	Preferred Reporting Items for Systematic Reviews and Meta-Analyses
RTA	Road Traffic Accidents
SD	Standard deviation
TAF	Tibial Anterior Cruciate Ligament Avulsion Fractures
