# Supplementary material for: Tibial Anterior Cruciate Ligament Avulsion Fractures in Pediatric and Adult Populations: A Systematic Literature Review
Source: J Clin Med. 2025 Sep 7;14(17):6316. doi: 10.3390/jcm14176316 (PMC12429662; doi:10.3390/jcm14176316)
Supplement: Supplementary file 1 [file jcm-14-06316-s001.zip › jcm-3801358-supplementary.pdf]

Systematic Review / Eminentia Frakturen → Versorgungsstrategien

PEC(C)O Question

Concept	
Patient, Population, Problem	All
Exposure	Fractures Eminentia intercondylaris
Outcome	Treatment

FINAL:

"Anterior Cruciate Ligament"[MeSH Terms] OR "ACL"[All Fields] OR "Anterior Cruciate Ligament"[All Fields] OR "Eminentia intercondylaris"[All Fields] OR "intercondyloid eminence"[All Fields] OR "intercondylar eminence"[All Fields] OR "tibial eminence"[All Fields] OR "tibial spine"[All Fields]

AND

"fractures, bone"[MeSH Terms] OR "fractures, avulsion"[MeSH Terms] OR "fracture"[All Fields] OR "fracture*"[All Fields] OR "avulsion"[All Fields] OR "avulsion*"[All Fields]

AND

(2000:2023[pdat])

History and Search Details

Download Delete

Search	Actions	Details	Query	Results	Time
#4	•••	>	Search: **#1 AND #2 AND #3**	1,518	04:57:34
#3	•••	>	Search: **(2000:2023[pdat])**	21,951,664	04:57:22
#2	•••	>	Search: **"fractures, bone"[MeSH Terms] OR "fractures, avulsion"[MeSH Terms] OR "fracture"[All Fields] OR "fracture*"[All Fields] OR "avulsion"[All Fields] OR "avulsion*"[All Fields]**	366,214	04:57:17
#1	•••	>	Search: **"Anterior Cruciate Ligament"[MeSH Terms] OR "ACL"[All Fields] OR "Anterior Cruciate Ligament"[All Fields] OR "Eminentia intercondylaris"[All Fields] OR "intercondyloid eminence"[All Fields] OR "intercondylar eminence"[All Fields] OR "tibial eminence"[All Fields] OR "tibial spine"[All Fields]**	34,650	04:57:12

Showing 1 to 4 of 4 entries

EMBASE

'anterior cruciate ligament injury'/exp OR 'ACL':ab,ti OR 'anterior cruciate ligament':ab,ti OR 'eminentia intercondylaris':ab,ti OR 'intercondyloid eminence':ab,ti OR 'intercondylar eminence':ab,ti OR 'tibial eminence':ab,ti OR 'tibial spine':ab,ti

AND

'fracture'/exp OR 'avulsion injury'/exp OR 'fracture':ab,ti OR 'fracture*':ab,ti OR 'avulsion':ab,ti OR 'avulsion*':ab,ti

AND

[2000-2024]/py

History | Save | Delete | Print view | Export | Email | Combine | using And / Or | Collapse

#4	#1 AND #2 AND #3	2,413
#3	[2000-2023]/py	28,044,508
#2	'fracture'/exp OR 'avulsion injury'/exp OR 'fracture':ab,ti OR 'fracture*':ab,ti OR 'avulsion':ab,ti OR 'avulsion*':ab,ti	487,289
#1	'anterior cruciate ligament injury'/exp OR 'acl':ab,ti OR 'anterior cruciate ligament':ab,ti OR 'eminentia intercondylaris':ab,ti OR 'intercondyloid eminence':ab,ti OR 'intercondylar eminence':ab,ti OR 'tibial eminence':ab,ti OR 'tibial spine':ab,ti	38,808

Overview:
Search conducted 12/2024
Imported in Endnote: 3931
    Export Pubmed: 1518
    Export EMBASE: 2413
    Dublicates erased: 1070
Remaining: 2861 → import Rayyan

Duplicates removed in Rayyan: 161
Remaining for abstract screening: 2700
Included after abstract screening: 217
